# Extent of resection and molecular pathologic subtype are potent prognostic factors of adult WHO grade II glioma

**DOI:** 10.1038/s41598-020-59089-x

**Published:** 2020-02-07

**Authors:** Jinhyun Choi, Se Hoon Kim, Sung Soo Ahn, Hye Jin Choi, Hong In Yoon, Jae Ho Cho, Tae Hoon Roh, Seok-Gu Kang, Jong Hee Chang, Chang-Ok Suh

**Affiliations:** 1 Department of Radiation Oncology, Jeju National University Hospital, Jeju, Korea; 2 Department of Pathology, Yonsei University College of Medicine, Seoul, Korea; 3 Department of Radiology, Yonsei University College of Medicine, Seoul, Korea; 4 Department of Internal Medicine, Yonsei University College of Medicine, Seoul, Korea; 5 Department of Radiation Oncology, Yonsei University College of Medicine, Seoul, Korea; 6 Department of Neurosurgery, Ajou University School of Medicine, Suwon, Korea; 7 Department of Neurosurgery, Yonsei University College of Medicine, Seoul, Korea; 8 Department of Radiation Oncology, CHA Bundang Medical Center, CHA University, Seongnam, Korea

**Keywords:** Medical research, Molecular medicine, Oncology

## Abstract

We evaluated prognostic factors of adult low-grade glioma (LGG) according to the new 2016 WHO classification. Records of 153 patients diagnosed with WHO grade II LGG between 2003 and 2015 were retrospectively reviewed. Based on the 2016 WHO classification, 80 patients (52.3%) had diffuse astrocytoma, *IDH*-mutant; 45 (29.4%) had oligodendroglioma, *IDH*-mutant and 1p/19q-codeleted (ODG); and 28 (18.3%) had diffuse astrocytoma, *IDH*-wildtype. Gross total resection (GTR) was performed in 71 patients (46.4%), subtotal resection in 31 (20.3%), partial resection in 43 (28.1%), and biopsy in 8 (5.2%). One hundred two patients (66.7%) received postoperative radiotherapy. The 5- and 10-year progression-free survival (PFS) rates were 72.7% and 51.5%, respectively, and the 5- and 10-year overall survival (OS) rates were 82.5% and 63.5%, respectively. GTR and *IDH*-mutant and/or 1p/19q codeletion were favorable prognostic factors for PFS and OS. Patients with *IDH*-wildtype had significantly decreased OS. Among patients with ODG who underwent GTR, no recurrence was observed after radiotherapy. Patients who underwent non-GTR frequently experienced recurrence after radiotherapy (*IDH*-mutant: 47.6%, *IDH*-wildtype: 57.9%). In conclusion, molecular classification of LGG was of prognostic relevance, with *IDH*-wildtype patients having a particularly poor outcome, regardless of the treatment. Favorable results were observed in patients who underwent GTR.

## Introduction

World Health Organization (WHO) grade II low-grade glioma (LGG) included astrocytoma, oligodendroglioma, and oligoastrocytoma^1^. LGG has relatively favorable clinical outcomes and a slow progression without serious neurologic symptoms^2^.

With increasing evidence that molecular markers, such as isocitrate dehydrogenase (*IDH*) 1/2 gene mutation and chromosome 1p/19q codeletion, are more important than histologic subtype in the prediction of tumor response to treatment and prognosis, phenotypic and genotypic parameters have been integrated in the updated 2016 WHO classification of gliomas^3–6^. As most previous studies about prognostic factors in LGG were based on the old histologic classification, the impact of prognostic factors in the different molecular subtypes remains to be determined. Although maximal safe resection followed by adjuvant radiotherapy (RT) and PCV (procarbazine, lomustine, and vincristine) or temozolomide for high-risk patients (≤40 year old or subtotal resection)^7^ is the current standard of care, optimal treatment for each molecular subtype of LGG remains disputed.

This study aimed to validate the molecular pathologic subtypes as prognostic factors. Also, we examine the impact of surgery and adjuvant RT on outcomes in molecularly defined LGG.

## Methods

### Patient cohort and treatment

A total of 153 adult patients with pathologically confirmed WHO grade II LGG treated at Yonsei University Health System between March 2003 and November 2015 were retrospectively evaluated. In our institution, we aim for maximal safe surgical resection in patients with neurologic symptoms and suspected LGG in MRI. All patients underwent MRI before surgery and within 48 hours after resection. After surgical resection, radiotherapy and/or chemotherapy were performed. Although postoperative RT for patients with subtotal resection (STR) or age 40 years or over is standard in our institution, it was not strictly applied in this cohort due to the treating oncologists’ discretion and the patients’ preference. RT was performed with 3D conformal RT or intensity-modulated RT. The RT dose was 40–60 Gy (median 50.4 Gy), with 1.8–2 Gy per fraction. The RT volume was the surgical bed and T2 (or FLAIR) hyperintensity on postoperative MRI plus a 1.5–2-cm margin. The study protocol conformed to the ethical guidelines of the 1975 Declaration of Helsinki, as revised in 1983, and approved by the Institutional Review Board of Severance Hospital (No. 4-2016-0358), with a waiver of informed consent. This was a retrospective study for which all data were kept anonymous.

### Molecular pathologic parameters and surgical resection assessment by MRI

Molecular parameters, such as the deletion of 1p/19q, mutation of *IDH*1/2, or O6-methylguanine-DNA methyltransferase (*MGMT*) promotor methylation, were reviewed, and a pathologist revised the diagnosis using the 2016 WHO classification. The 1p19q status was examined in all gliomas from 2009 until the present, *MGMT* from 2010 until the present, and *IDH* from 2013 until the present. For earlier cases, paraffin blocks of tissue taken at the time of surgery were obtained and used for retrospective examination.

We examined *IDH1* mutations using the Ventana Bench Mark XT autostainer (Ventana Medical System, Inc., Tucson, AZ, USA) according to the protocol as described at our previous report^8^. The anti-human *IDH1* R132H mouse monoclonal antibody was used (Clone H09L, 1:80 dilution; Dianova, Hamburg, Germany). When the cytoplasmic expression of *IDH1* R132H was identified in glioma cells, we considered the case as “mutant”/“positive.” Fluorescent *in situ* hybridization analysis of the 1p/19q status was performed using the LSI 1p36/1q25 and 19q13/19p13 Dual-Color Probe Kit (Abbott Molecular Inc., Abbott Park, IL, USA). If the numbers of “deleted” nuclei exceeded 50%, the tumor was considered to show a “deletion” of the targeted chromosome^9^. *MGMT* gene promoter methylation was assessed by methylation-specific polymerase chain reaction^10^.

We classified the EOR according to the volume of the removed tumor measured on postoperative T2-weighted MR images by a neuroradiologist and operation records by a neuro-surgeon, and EOR was defined as gross total resection (GTR) when >99% of the tumor was removed, STR when 90–99% was removed, partial resection (PR) when 50–90% was removed, and biopsy when <50% was removed. When a discrepancy between postoperative MRI and operation findings was identified, a larger residual tumor volume was chosen as EOR for analysis.

### Statistical analysis

Progression free-survival (PFS) and overall survival (OS) were measured from the date of pathologically confirmed LGG to the date of documented progression and death or the last follow-up, respectively. Recurrence or progression of disease was defined with follow-up MRI according to the response assessment using the RANO criteria^11^. In terms of the RT field, in-field and out-field failure were defined as disease inside and outside the treatment field, respectively. Survival data were analyzed using the Kaplan-Meier method, and comparisons were performed using a two-sided log-rank test. Multivariate analyses were performed using the Cox proportional hazard regression model. The criteria for the inclusion of variables in a multivariate analysis were statistical significance in the univariate analysis and clinical relevance. Statistical analyses were performed with SPSS version 20.0 (IBM Corp., Armonk, NY, USA). A p-value ≤ 0.05 was considered statistically significant.

## Results

### Patient/tumor characteristics

**Figure 1:**
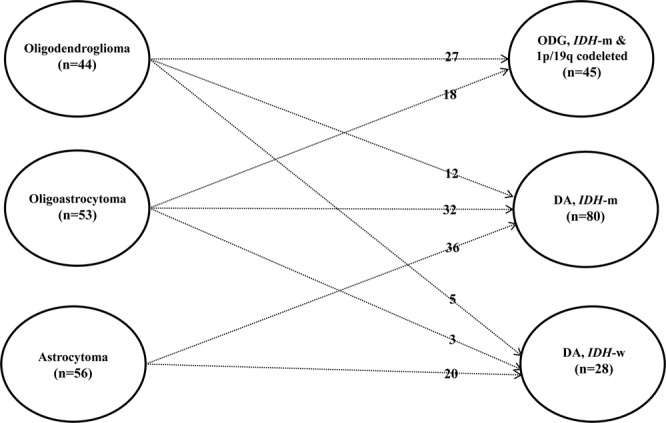
Patients’ distribution from histopathologic subtypes to molecular subtypes according to the new 2016 WHO classification.

The median age at diagnosis was 41 years (range, 22–74 years). The initial pathologic diagnosis was astrocytoma in 56 patients, oligodendroglioma in 44 patients, and oligoastrocytoma in 53 patients. Based on the 2016 WHO classification, 45 (29.4%) patients had oligodendroglioma, *IDH*-mutant and 1p/19q codeleted (ODG); 80 (52.3%) had diffuse astrocytoma, *IDH*-mutant (IDHmt); and 28 (18.3%) had diffuse astrocytoma, *IDH*-wildtype (IDHwt). Figure 1 shows the change in distribution from histopathologic subtypes to molecular subtypes when the new 2016 WHO classification was applied.

GTR was performed in 71 patients (46.4%), STR in 31 (20.3%), PR in 43 (28.1%), and biopsy in 8 (5.2%). The GTR rate was lower in the IDHwt (32.1%) group than in the IDHmt (47.5%) and ODG (53.3%) groups. In 44 patients (28.8%) in whom tumors were near the parts of the brain that control vision, movement, or speech, awake brain surgery was performed. The frontal lobe was the most commonly involved lobe (53.6%). Most patients had a tumor size <6 cm (70.6%). One hundred and two patients (66.7%) received postoperative RT, 38 (24.8%) received adjuvant chemotherapy, and 26 (17%) received both RT and chemotherapy. RT was administered more frequently in patients with poor prognostic factors such as a larger tumor size and/or non-GTR, as shown in Supplementary Table 1. As the molecular subtype was not considered when selecting adjuvant RT during this study period, the proportions of IDHwt patients in the RT and non-RT groups were similar (19.6% vs. 15.6%). The majority of patients treated with chemotherapy (n = 34) received temozolomide-based treatment, and the remaining patients received lomustine. Detailed information regarding the clinical characteristics according to molecular pathologic subtype is presented in Table 1. Age and tumor location significantly differed based on molecular subtype. Patients with IDHmt were younger than patients with other subtypes. ODG more frequently involved the frontal lobe, and three-fourths of IDHwt cases involved non-frontal lobes. There was no difference in sex, tumor size, EOR, adjuvant treatment, or *MGMT* methylation status among the groups.

**Table 1:** Patients’ characteristics.

Variable	Level	N (%)	ODG (n = 45, %)	DA, IDH-m (n = 80, %)	DA, IDH-w (n = 28, %)	*p* value
Age	<40 yr	66 (43)	16 (36)	44 (55)	6 (21)	0.004
	≥40 yr	87 (57)	29 (64)	36 (45)	22 (79)	
Sex	M	86 (56)	23 (51)	49 (61)	14 (50)	0.419
	F	67 (44)	22 (49)	31 (39)	14 (50)	
Tumor size	<6 cm	108 (71)	31 (69)	58 (73)	19 (68)	0.856
	≥6 cm	42 (27)	14 (31)	21 (26)	7 (25)	
Tumor location	Frontal lobe	82 (54)	30 (67)	45 (56)	7 (25)	0.001
	Non-frontal lobe	71 (46)	15 (33)	35 (44)	21 (75)	
EOR	GTR	71 (46)	24 (53)	38 (48)	9 (32)	0.202
	Non-GTR	82 (54)	21 (47)	42 (52)	19 (68)	
RT	Yes	102 (67)	26 (58)	56 (70)	20 (71)	0.318
	No	51 (33)	19 (42)	24 (30)	8 (29)	
CTx	Yes	38 (25)	15 (33)	18 (23)	5 (18)	0.258
	No	115 (75)	30 (67)	62 (77)	23 (82)	
RT + CTx	Yes	26 (17)	8 (18)	14 (17)	4 (14)	0.914
	No	127 (83)	37 (82)	66 (83)	24 (86)	
MGMT	Methylation	111 (73)	40 (89)	63 (79)	8 (29)	0.869
	Unmethylation	40 (26)	5 (11)	17 (21)	18 (64)	

Abbreviations: ODG, oligodendroglioma; DA, diffuse astrocytoma; EOR, extent of resection; GTR, gross total resection; RT, radiotherapy; CTx, chemotherapy; Tx, treatment; MGMT, O6-methylguanine-DNA methyltransferase promotor.

### Treatment outcomes and prognostic factors

**Figure 2:**
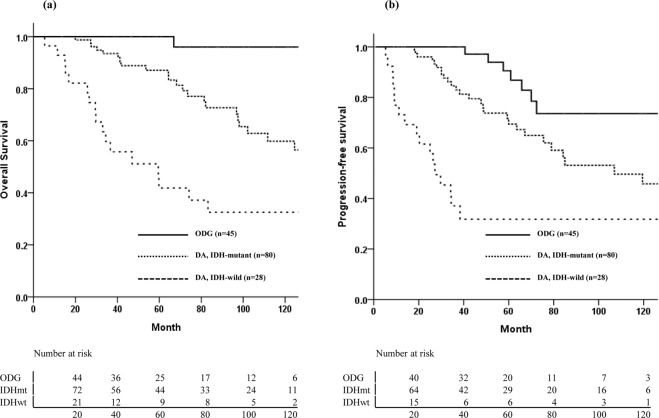
Overall survival (**a**) and progression-free survival (**b**) according to molecular subtype.

The median follow-up time was 66.9 months (range, 5.3–171.3 months). Disease progression or recurrence was defined as treatment failure. During the follow-up period, 49 patients (32%) experienced treatment failures, including 7 in the ODG group (15.6%), 27 in the IDHmt group (33.8%), and 15 in the IDHwt group (53.6%). The median times to treatment failure were 60.9 months in the ODG group, 42.3 months in the IDHmt group, and 19.1 months in the IDHwt group. Overall, 42 patients died, and the median follow-up period of the 111 survivors was 67.1 months. The 5- and 10-year PFS rates were 72.7% and 51.5%, respectively, and the 5- and 10-year OS rates were 82.5% and 63.5%, respectively (Supplementary Fig. 1). The 10-year OS rates for the ODG, IDHmt, and IDHwt groups were 96%, 59.8%, and 32.5%, respectively (p < 0.001, Fig. 2a). The corresponding 10-year PFS rates were 93.6%, 45.8%, and 31.8%, respectively (p < 0.001, Fig. 2b). Prognostic factor analysis was performed using the variables listed in Table 2. GTR, molecular subtype of *IDH*-mutant and/or 1p/19q codeletion, and tumor size less than 6 cm were favorable prognostic factors for both PFS and OS. Adjuvant RT was correlated with poor OS. Chemotherapy was an independent prognostic factor only for PFS. Multivariate analysis showed that molecular pathologic subtype and EOR were both significant factors for OS and PFS. The molecular markers had significance as an independent prognostic factor, and statistical significance was also shown in separate survival analyses of 1p/19q codeletion, *IDH* mutation, and *MGMT* methylation status (Supplementary Fig. 2).

**Table 2:** Univariate and multivariate analyses of predictors of overall and progression-free survival in low-grade glioma patients.

Variable	N (%)	10yr-OS	Univariate	Multivariate			10yr-PFS	Univariate	Multivariate		
		(%)	p	HR	95% CI	p	(%)	p	HR	95% CI	p
Age (yr)			0.012					0.881			
<40	66 (43)	65.6					63.2				
≥40	87 (57)	58.5					42.8				
Molecular subtype			<0.001			<0.001		<0.001			<0.001
DA, IDH-mutant	80 (52)	59.8		6.68	1.56–28.52		45.8		2.45	1.05–5.69	
ODG	45 (29)	96		*			73.6		*		
DA, IDH-wild	28 (18)	36.1		24.66	5.58–108.98		32.5		9.09	3.6–22.94	
Tumor size (cm)			0.012			0.043		0.054			0.044
<6	108 (71)	71.7		*			56.5		*		
≥6	42 (27)	48.7		1.98	1.03–3.81		38.2		1.94	1.03–3.64	
EOR			<0.001			<0.001		0.003			0.009
GTR	71 (46)	80.6		*			66.1		*		
Non-GTR	82 (54)	50.9		3.84	1.73–8.53		41.3		2.24	1.21–4.52	
Tumor location			0.004					0.001			
Frontal	82 (54)	70.1					60.6				
Non-frontal	71 (46)	55.2					40.4				
RT			0.006					0.403			
Yes	102 (67)	57.1					50.8				
No	51 (33)	74.4					54				
CTx			0.098					0.019			0.011
Yes	38 (25)	76.6					66.2		*		
No	115 (75)	55.6					43.7		2.49	1.17–5.28	

Abbreviations: DA, diffuse astrocytoma; ODG, oligodendroglioma; EOR, extent of resection; GTR, gross total resection; Tx, treatment; RT, radiotherapy; CTx, chemotherapy; OS, overall survival; PFS, progression-free survival; IDH, isocitrate dehydrogenase; *Reference category.

### Patient outcomes according to the molecular subtype and treatment modalities

**Figure 3:**
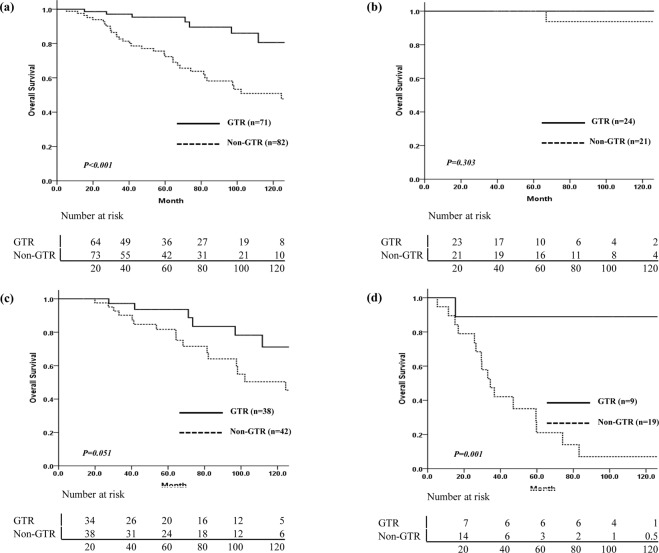
Comparison of overall survival in all patients and each molecular subtype by extent of resection. (**a**) Overall, (**b**) ODG, (**c**) IDHmt, (**d**) IDHwt. ODG: oligodendroglioma, isocitrate dehydrogenase-mutant and 1p/19q codeleted; IDHmt: diffuse astrocytoma, isocitrate dehydrogenase-mutant; IDHwt: diffuse astrocytoma, isocitrate dehydrogenase-wild-type.

**Figure 4:**
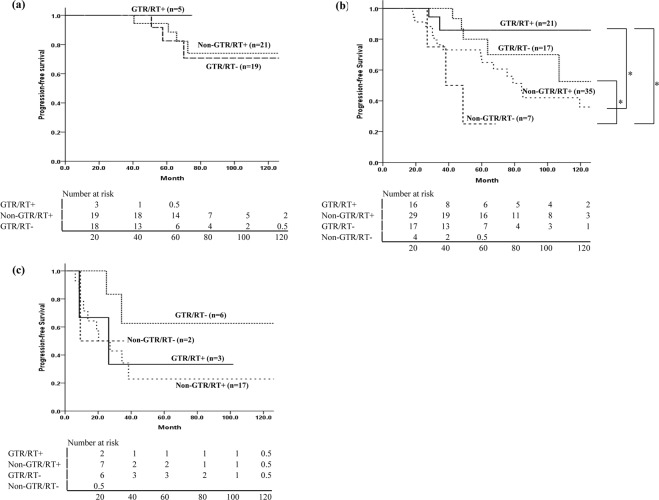
Analysis of progression-free survival according to extent of resection and use of radiotherapy in each molecular subtype. (**a**) ODG, (**b**) IDHmt, (**c**) IDHwt. *Means statistically significant difference in the two groups. ODG: oligodendroglioma, isocitrate dehydrogenase-mutant and 1p/19q codeleted; IDHmt: diffuse astrocytoma, isocitrate dehydrogenase-mutant; IDHwt: diffuse astrocytoma, isocitrate dehydrogenase-wild-type.

Treatment failures occurred in 14/71 (19.7%) patients who underwent GTR and 35/82 (42.7%) patients who received non-GTR. The EOR affected the survival outcomes in the IDHmt and IDHwt groups, but not in the ODG group (Fig. 3). Among 21 patients with ODG who received non-GTR and postoperative RT, 4 showed progression at a range of 40.6–72.4 months after surgery, and only 1 patient died of the disease, 66.9 months after diagnosis. In the IDHmt group, the OS difference according to the EOR was marginally significant (p = 0.051). Among 19 patients with IDHwt who had non-GTR (17 patients received postoperative RT), 11 showed progression at a median 13.8 months (range, 6.2–38.4 months) after surgery, and the median survival time was 33 months. Analysis of PFS according to the EOR and use of RT in each molecular subtype did not show any significant difference (Fig. 4). In the IDHmt group, the 10-year PFS of 21 patients who received GTR and postoperative RT was better than that of 17 patients who received GTR without RT, but this was not statistically significant (85.9% vs. 52.5%, p = 0.469). Among 5 patients with ODG who underwent GTR, no recurrence was observed after RT without chemotherapy. In contrast, among patients in the IDHwt group, the PFS was poor irrespective of the EOR or administration of RT (Fig. 4-c, Supplementary Table 2).

## Discussion

In this study, we confirmed that molecular pathologic subtype based on the 2016 WHO classification was a very powerful prognostic factor in this Korean patient cohort. In addition, we observed that the EOR affected the survival outcome. As the treatment policy of our institution has been maximal safe surgical resection, the proportion of GTR was higher than that in other studies, which had a 4–15% GTR rates^12–14^. Consequently, we obtained relatively high survival rates in all three molecular subtypes as compared to those in other series.

The ODG group showed excellent outcomes. Although only one-fourth of patients received adjuvant chemotherapy, the 10-year OS rate of ODG patients was 96%, likely because of the high rate of GTR (53%) and because all 21 patients with non-GTR received RT. As compared with the IDHmt group, survival outcomes in the IDHwt group were poor, similar to those in patients with anaplastic astrocytoma, *IDH*-wildtype (5-year OS: 47.5%) and worse than those in patients with anaplastic astrocytoma, *IDH*-mutant (5-year OS: 71.6%) in our previous study^8^. As grade II gliomas and grade III gliomas share molecular-genetic markers that are stronger prognostic factors than histologic grade, WHO grade II and III gliomas are now categorized together as “lower-grade gliomas”. *IDH*-wildtype tumors are clinically similar to glioblastoma and are called as the glioblastoma-like subtype^14^.

Although there has been no prospective randomized trial to assess the role of the EOR in LGGs, several retrospective studies showed that increasing the amount of tumor removal was significantly correlated with improvement in both PFS and OS. Currently, maximal safe resection is recommended^15,16^. The feasibility of maximal surgical resection differs based on molecular subtypes and tumor location; both are correlated. *IDH*-mutant tumors are more frequently located in the frontal and temporal lobes, which are more amenable to resection. In this study, *IDH*-wildtype tumors were more commonly located in the non-frontal area and had a lower GTR rate (32%). The impact of the EOR on prognosis in each molecular subtype has not been well studied. In high-grade gliomas, the impact of residual tumor on survival differs between *IDH1*-wildtype and *IDH1*-mutant tumors^17^. *IDH1*-mutant tumors have an additional survival benefit associated with maximal resection, but in *IDH1*-wildtype tumors, no survival benefit is observed in association with further resection of residual tumor. The impact of surgery in molecularly defined LGG was evaluated retrospectively by Wijnenga *et al*.^14^. Postoperative tumor volume was associated with OS, and the impact of postoperative volume was particularly strong in *IDH*-mutant tumors. They concluded that maximal safe resection is important in *IDH*-mutated astrocytoma and argued for a second-look operation to remove minor residue in this subtype. They also observed that the impact of small residual tumor volume was not strong in oligodendroglioma, which was interpreted as being due to its more indolent nature and increased sensitivity to treatment. As about half of the patients in this study had GTR, we dichotomized the EOR into GTR vs. non-GTR. Although our patient cohort was small and adjuvant therapy was heterogeneous, non-GTR patients showed earlier progression and poorer survival than GTR patients in both the IDHmt and IDHwt groups. In the ODG group, the survival difference according to the EOR was not significant. All the patients in the ODG group with non-GTR received postoperative RT, and there were few recurrences in both the non-GTR with RT group and the GTR without RT group. In the IDHwt group, the impact of GTR was prominent, but patients with GTR had better prognostic factors, such as younger age, small tumor size (2.2–6.7 cm, median 2.9 cm), and non-eloquent area location.

The optimal postoperative adjuvant therapy for LGG has long been a controversial issue. Furthermore, with the introduction of molecular subtypes in the clinic, we need more information about the efficacy of RT or chemotherapy in each molecular subtype. Traditionally, in patients who are considered low-risk, defined as those younger than 40 years with GTR, regular follow-up without adjuvant treatment is recommended because of the indolent nature of the disease and the risk of late complications of radiotherapy. In a large prospective study, however, 52% of patients with low-risk LGG presented with recurrence within 5 years of surgery^18^. The RTOG and EORTC trials have evaluated the role of radiation therapy, including dose escalation and the timing of RT. Collectively, these trials showed no survival benefit with a higher RT dose, but did demonstrate a significant benefit in PFS (5.3 years vs. 3.4 years) for patients undergoing early radiation therapy compared to those undergoing delayed radiation therapy^19–21^. As we administered adjuvant RT to high-risk patients, the survival outcomes were worse in patients who received RT than in those who did not receive RT.

To determine the role of RT in each molecular subtype, we performed subgroup analysis. In ODG patients, the patients with GTR without RT and those with non-GTR and RT had similar survival outcomes, suggesting an effect of RT. The fact that no recurrence occurred in the patients who received GTR and RT could be criticized as overtreatment. Proper management after GTR, whether observation, RT or chemotherapy, should be evaluated in terms of survival, neurocognitive function, and quality of life^22^. A prospective study administering postoperative temozolomide for 1 year showed that patients with 1p/19q codeletion demonstrated a 0% risk of progression during treatment, and the median PFS and OS rates of patients with 1p/19q-codeleted tumors were 4.2 and 9.7 years, respectively^23^. However, the choice of temozolomide over radiotherapy alone in patients with high-risk LGG is not supported by the evidence. The EORTC study to evaluate health-related quality of life in patients with high-risk LGG showed no difference between temozolomide chemotherapy and radiotherapy^24^. Although a randomized trial for high-risk LGG (RTOG 9802) showed that patients who received RT plus PCV had a longer median OS than those who received RT alone (13.3 vs. 7.8 years), only 10% of the patients received GTR, and oligodendroglioma patients were not separately analyzed. The efficacy of postoperative adjuvant therapy for ODG patients, whether temozolomide alone, RT alone, or RT followed by chemotherapy, still requires proper evaluation.

In IDHmt patients with GTR, better PFS was observed with RT, although it was not statistically significant, probably due to the small number of patients (Fig. 4b). *IDH*-mutant tumors have been shown to have higher sensitivity to radiation experimentally and clinically^25^. Additionally, most *IDH*-mutant tumors have *MGMT* promotor methylation, which increases radiosensitivity by inhibiting DNA repair^26^. In a preliminary analysis of an ongoing clinical trial to compare temozolomide versus RT for high-risk LGG (EORTC 22033-26033), patients with *IDH*-mutant/non-codeleted tumors treated with RT had a longer PFS than those treated with temozolomide^13^. As the IDHwt subgroup was small (n = 28) in the current study, it was difficult to find any difference according to RT administration.

The limitations of this study were its retrospective nature and the small number of patients. Moreover, heterogeneity in adjuvant therapy could have affected the survival outcomes and hindered the evaluation of the role of RT or chemotherapy. Adjuvant RT was administered more frequently in patients with worse prognostic factors, which confused the role of RT. In the current study, only one-fourth of the patients received chemotherapy because there was no consensus regarding the use of chemotherapy for LGG during the study period and because the medical expenses related to chemotherapy were not reimbursed by the National Health Insurance of our country. Therefore, these findings regarding the role of adjuvant therapy have limited generalizability. However, we assessed EOR in all patients using postoperative MRI, which is the most important test to guide treatment decisions.

In conclusion, molecular pathologic subtype of LGG as defined in the new 2016 WHO classification was of prognostic relevance. Patients with tumors that did not have *IDH* mutations had a particularly poor outcome, regardless of adjuvant treatment, and ODG patients showed excellent long-term survival. Favorable results were observed in patients who had undergone GTR. Postoperative RT might have a role in improving survival in patients with *IDH*-mutant tumors. We suggest that clinical trials assessing the efficacy of adjuvant therapy for LGG should be stratified by molecular subtype and EOR.

## Supplementary information

Supplementary information.

Supplementary information 2.

Supplementary information 3.

## Data Availability

All relevant data are within the paper.
