# Supplementary material for: Extent of resection and molecular pathologic subtype are potent prognostic factors of adult WHO grade II glioma
Source: Sci Rep. 2020 Feb 7;10:2086. doi: 10.1038/s41598-020-59089-x (PMC7005814; doi:10.1038/s41598-020-59089-x)
Supplement: Supplementary file 1 — Supplementary information. [file 41598_2020_59089_MOESM1_ESM.pdf]

Extent of resection and molecular pathologic subtype are potent prognostic factors of adult WHO grade II glioma

Jinhyun Choi, MD,[1] Se Hoon Kim, MD, PhD,[2] Sung Soo Ahn, MD, PhD,[3] Hye Jin Choi, MD, PhD,[4] Hong In Yoon, MD, PhD,[5] Jae Ho Cho, MD, PhD,[5] Tae Hoon Roh, MD, PhD,[6] Seok-Gu Kang, MD, PhD,[7] Jong Hee Chang, MD, PhD,[7*] and Chang-Ok Suh, MD, PhD[8*]

[1]Department of Radiation Oncology, Jeju National University Hospital, Jeju, Korea

[2]Department of Pathology, Yonsei University College of Medicine, Seoul, Korea

[3]Department of Radiology, Yonsei University College of Medicine, Seoul, Korea

[4]Department of Internal Medicine, Yonsei University College of Medicine, Seoul, Korea

[5]Department of Radiation Oncology, Yonsei University College of Medicine, Seoul, Korea

[6]Department of Neurosurgery, Ajou University School of Medicine, Suwon, Korea

[7]Department of Neurosurgery, Yonsei University College of Medicine, Seoul, Korea

[8]Department of Radiation Oncology, CHA Bundang Medical Center, CHA University, Seongnam, Korea

Table S1 Patients' characteristics according to the use of radiotherapy

Variable	Level	RT (N=102)	%	No RT (N=51)	%
Tumor size	< 6 cm	64	62.7	44	86.3
	≥ 6 cm	35	34.3	7	13.7
	Unevaluable	3	2.9	0	0
EOR	GTR	29	28.4	42	82.4
	STR	23	22.5	8	15.7
	PR	42	41.2	1	1.9
	Bx	8	7.8	0	0

Abbreviations: RT, radiotherapy; EOR, extent of resection; GTR, gross total resection; STR, subtotal resection; PR, partial resection; Bx, biopsy

Table S2 Recurrence rate according to extent of resection and the use of radiotherapy

Molecular subtype	EOR	Radiotherapy	Recurrence rate (%)		
ODG, IDH-mutant & 1p/19q codeleted	GTR (24)	RT (5)	0	12.5	15.6
		no RT (19)	15.8		
	Non-GTR (21)	RT (21)	19	19	
		no RT (0)	-		
DA, IDH-mutant	GTR (38)	RT (21)	9.5	18.4	33.8
		no RT (17)	29.4		
	Non-GTR (42)	RT (35)	48.6	47.6	
		no RT (7)	42.9		
DA, IDH-wild	GTR (9)	RT (3)	66.7	44.4	53.6
		no RT (6)	33.3		
	Non-GTR (19)	RT (17)	58.8	57.9	
		no RT (2)	50		

Numbers in parentheses are the numbers of patients

Abbreviations: ODG, oligodendroglioma; DA, diffuse astrocytoma; EOR, extent of resection; GTR, gross total resection; RT, radiotherapy
