# Supplementary material for: Extent of resection and molecular pathologic subtype are potent prognostic factors of adult WHO grade II glioma
Source: Sci Rep. 2020 Feb 7;10:2086. doi: 10.1038/s41598-020-59089-x (PMC7005814; doi:10.1038/s41598-020-59089-x)
Supplement: Supplementary file 2 — Supplementary information 2. [file 41598_2020_59089_MOESM2_ESM.pdf]

Extent of resection and molecular pathologic subtype are potent prognostic factors of adult WHO grade II glioma

Jinhyun Choi, MD,[1] Se Hoon Kim, MD, PhD,[2] Sung Soo Ahn, MD, PhD,[3] Hye Jin Choi, MD, PhD,[4] Hong In Yoon, MD, PhD,[5] Jae Ho Cho, MD, PhD,[5] Tae Hoon Roh, MD, PhD,[6] Seok-Gu Kang, MD, PhD,[7] Jong Hee Chang, MD, PhD,[7*] and Chang-Ok Suh, MD, PhD[8*]

[1]Department of Radiation Oncology, Jeju National University Hospital, Jeju, Korea
[2]Department of Pathology, Yonsei University College of Medicine, Seoul, Korea
[3]Department of Radiology, Yonsei University College of Medicine, Seoul, Korea
[4]Department of Internal Medicine, Yonsei University College of Medicine, Seoul, Korea
[5]Department of Radiation Oncology, Yonsei University College of Medicine, Seoul, Korea
[6]Department of Neurosurgery, Ajou University School of Medicine, Suwon, Korea
[7]Department of Neurosurgery, Yonsei University College of Medicine, Seoul, Korea
[8]Department of Radiation Oncology, CHA Bundang Medical Center, CHA University, Seongnam, Korea

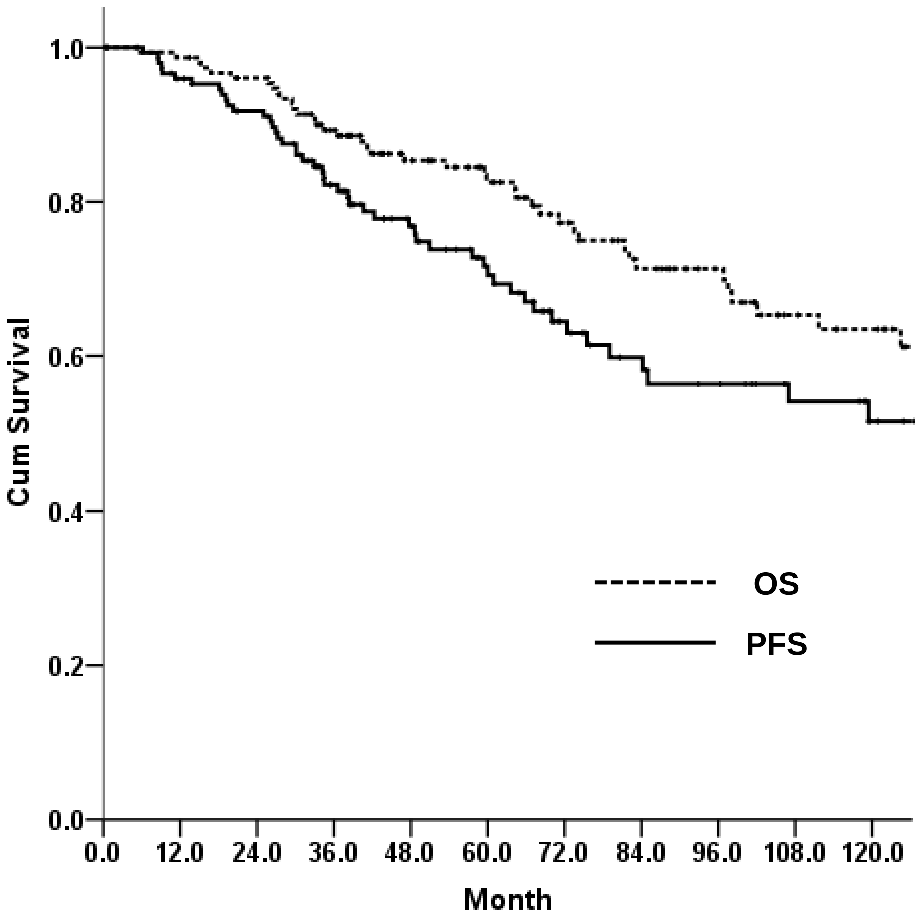

Number at risk

OS	150	140	115	94	79	66	54	45	35	18
PFS	138	122	93	74	57	42	35	28	22	9
	12	24	36	48	60	72	84	96	108	120
