# Supplementary material for: Extent of resection and molecular pathologic subtype are potent prognostic factors of adult WHO grade II glioma
Source: Sci Rep. 2020 Feb 7;10:2086. doi: 10.1038/s41598-020-59089-x (PMC7005814; doi:10.1038/s41598-020-59089-x)
Supplement: Supplementary file 3 — Supplementary information 3. [file 41598_2020_59089_MOESM3_ESM.pdf]

Extent of resection and molecular pathologic subtype are potent prognostic factors of adult WHO grade II glioma

Jinhyun Choi, MD,[1] Se Hoon Kim, MD, PhD,[2] Sung Soo Ahn, MD, PhD,[3] Hye Jin Choi, MD, PhD,[4] Hong In Yoon, MD, PhD,[5] Jae Ho Cho, MD, PhD,[5] Tae Hoon Roh, MD, PhD,[6] Seok-Gu Kang, MD, PhD,[7] Jong Hee Chang, MD, PhD,[7*] and Chang-Ok Suh, MD, PhD[8*]

[1]Department of Radiation Oncology, Jeju National University Hospital, Jeju, Korea
[2]Department of Pathology, Yonsei University College of Medicine, Seoul, Korea
[3]Department of Radiology, Yonsei University College of Medicine, Seoul, Korea
[4]Department of Internal Medicine, Yonsei University College of Medicine, Seoul, Korea
[5]Department of Radiation Oncology, Yonsei University College of Medicine, Seoul, Korea
[6]Department of Neurosurgery, Ajou University School of Medicine, Suwon, Korea
[7]Department of Neurosurgery, Yonsei University College of Medicine, Seoul, Korea
[8]Department of Radiation Oncology, CHA Bundang Medical Center, CHA University, Seongnam, Korea

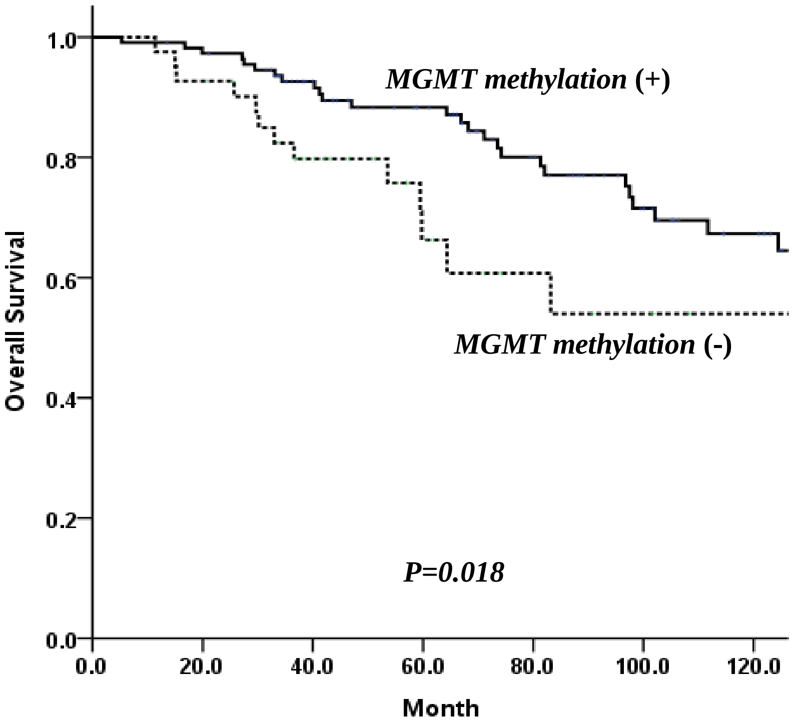

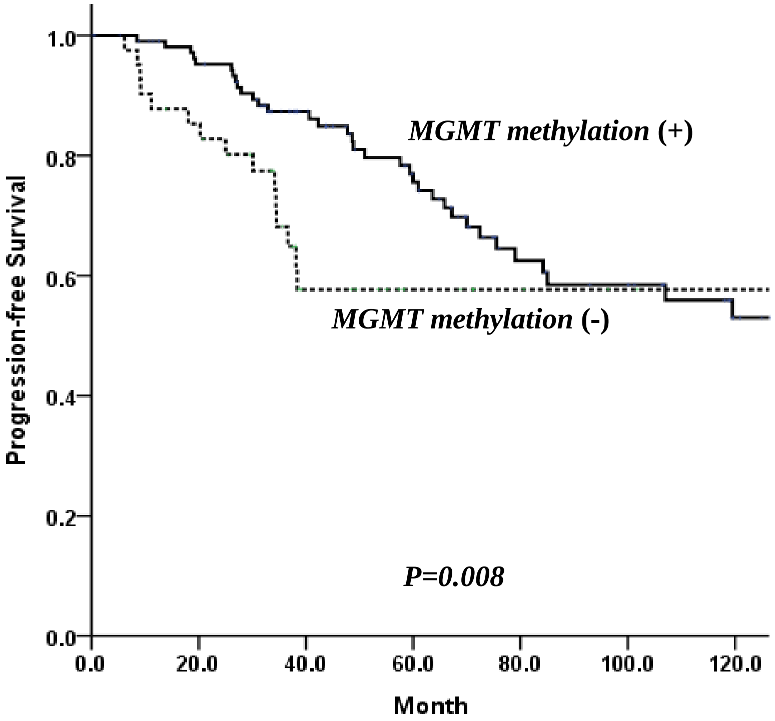

Number at risk

Methyl(+)	101	82	66	49	34	16
Methyl(-)	41	35	22	12	6	2
	20	40	60	80	100	120

Number at risk

Methyl(+)	90	68	48	31	23	8
Methyl(-)	29	11	7	4	3	1
	20	40	60	80	100	120
